# Association of Levels of Antibodies from Patients with Inflammatory Bowel Disease with Extracellular Proteins of Food and Probiotic Bacteria

**DOI:** 10.1155/2014/351204

**Published:** 2014-06-04

**Authors:** Arancha Hevia, Patricia López, Ana Suárez, Claudine Jacquot, María C. Urdaci, Abelardo Margolles, Borja Sánchez

**Affiliations:** ^1^Instituto de Productos Lácteos de Asturias, Consejo Superior de Investigaciones Científicas (IPLA-CSIC), Paseo Río Linares s/n, Villaviciosa, 33300 Asturias, Spain; ^2^Department of Functional Biology, Immunology Area, University of Oviedo, C/Julián Clavería s/n, Oviedo, 33006 Asturias, Spain; ^3^UMR 5248 CBMN CNRS-Université Bordeaux 1-ENITAB, Laboratoire de Microbiologie et Biochimie Appliquée, 1 Cours du Général de Gaulle, 33175 Gradignan Cedex, France; ^4^Nutrition and Bromatology Group, Department of Analytical and Food Chemistry, Food Science and Technology Faculty, University of Vigo, Ourense Campus, 32004 Ourense, Spain

## Abstract

Inflammatory bowel disease (IBD) is an autoimmune disease characterized by a chronic inflammation of the gastrointestinal tract mucosa and is related to an abnormal immune response to commensal bacteria. Our aim of the present work has been to explore the levels of antibodies (IgG and IgA) raised against extracellular proteins produced by LAB and its association with IBD. We analyzed, by Western-blot and ELISA, the presence of serum antibodies (IgA and IgG) developed against extracellular protein fractions produced by different food bacteria from the genera *Bifidobacterium* and *Lactobacillus*. We used a sera collection consisting of healthy individuals (HC, *n* = 50), Crohn's disease patients (CD, *n* = 37), and ulcerative colitis patients (UC, *n* = 15). Levels of IgA antibodies developed against a cell-wall hydrolase from *Lactobacillus casei* subsp. *rhamnosus* GG (CWH) were significantly higher in the IBD group (*P* < 0.002; *n* = 52). The specificity of our measurements was confirmed by measuring IgA antibodies developed against the CWH peptide 365-VNTSNQTAAVSAS-377. IBD patients appeared to have different immune response to food bacteria. This paper sets the basis for developing systems for early detection of IBD, based on the association of high levels of antibodies developed against extracellular proteins from food and probiotic bacteria.

## 1. Introduction

Inflammatory bowel disease (IBD) is an autoimmune disease characterized by a chronic inflammation of the gastrointestinal tract (GIT) mucosa. Depending on the severity and location of the injuries, two main forms are distinguished, Crohn's disease (CD) and ulcerative colitis (UC). Both are chronic disorders of unexplained origin, in which persistent ulcerations appear in the small or large bowel mucosa. Interestingly, genetic susceptibility only explains up to 23% of the disease, in the case of CD (16% for UC), with the rest being attributed to environmental factors, such as an exacerbated response of the innate immune system to the commensal microbiota [1].

Experiments in germ-free animals have shown that microbial colonization is crucial in the instruction, maturation, and regulation of the immune system. For instance, the presence of* Bacteroides fragilis* offers protection from experimental colitis, induced by* Helicobacter hepaticus*, in an animal model, with this beneficial activity being dependent on the presence of an exopolysaccharide [2]. In addition, recent metagenomic studies with human samples have revealed that lifestyle in developing countries is associated with an altered microbial colonization of the human gut [3]. Indeed, an altered microbial composition, or dysbiosis, is observed in both mucosal and fecal samples of patients with IBD [4].

Implications of microbiota dysbiosis extend beyond the obvious differences in microbial composition from a functional and metabolic point of view. For instance, decreased ratios between* Faecalibacterium prausnitzii *and* Escherichia coli* in IBD patients resulted in different fecal bile acid compositions, with implications in the perpetuation of chronic inflammation in IBD [5]. In the same way, Firmicutes and Enterobacteriaceae abundances have been related to changes in global metabolic pathways present in the gut microbiomes of IBD patients, notably with increases in oxidative stress pathways and decreases in carbohydrate metabolism and amino acid biosynthesis with respect to healthy controls [6]. For this reason, great efforts are being made in order to understand exactly the genome complement carried by all the microorganisms inhabiting our gut [7]. Although gut microorganisms are essential in driving inflammation and mucosal injuries in IBD, some other bacteria attenuate inflammation through anti-inflammatory effects, such as certain lactic acid bacteria (LAB) or the commensal bacterium* F. prausnitzii* [8–10]. Interestingly, LAB count with a long history of safe use by human beings [11, 12].

IBD is associated with antibodies raised against extracellular molecules of GIT microorganisms, such as extracellular mannan from* Saccharomyces cerevisiae* (ASCA), the outer membrane porin C protein from* E. coli* (anti-OmpC), or the flagellin from members of the* Clostridium *cluster XIVa [13–15]. The presence of these antibodies, together with some specific antibodies directed against host structures (such as antibodies to exocrine pancreas (PAB) or antineutrophil cytoplasmic antibodies (ANCA)), are used as serum biomarkers of CD or UC [13, 16].

Our aim with the present work has been to explore the levels of antibodies (IgG and IgA) raised against extracellular proteins produced by LAB and its association with IBD. The main results are discussed next.

## 2. Material and Methods

### 2.1. Culture Conditions and Bacterial Strains

Six bacterial strains representing microorganisms used in human nutrition, or as probiotics, were used in this study:* Lactobacillus casei* subsp.* rhamnosus* GG (LGG),* Lactobacillus acidophilus* DSM 20079^T^,* Lactobacillus reuteri* DSM 20016^T^,* Bifidobacterium longum* subsp.* longum* NCIMB 8809,* Bifidobacterium bifidum* LMG 11041^T^, and* Bifidobacterium animalis* subsp.* lactis* IPLA 4549. All strains were grown in MRS (de Man, Rogosa, and Sharpe) broth (Difco, Becton Dickinson, Franklin Lakes, NJ) supplemented with 0.05% (w/v) L-cysteine (MRSC) (Sigma-Aldrich, St. Louis, MO). Agar (1.8% (w/v)) was added to the broth when colony isolation was necessary. In all cases, cultures were incubated in an anaerobic chamber model MG500 (Don Whitley Scientific, West Yorkshire 100, UK) with a defined atmosphere composed of 10% (v/v) H_2_, 10% (v/v) CO_2_, and 80% N_2_.

As routine culturing, isolated bacterial colonies were recovered on MRSC agar plates from frozen stocks stored at −80°C (MRSC supplemented with 40% (v/v) glycerol). Single colonies were used for inoculating MRSC tubes, which were kept in the anaerobic chamber ON. Fresh MRSC bottles containing different volumes, depending on the experiment (from 50 mL to 400 mL), were then inoculated from the ON precultures (1% v/v). These cultures were incubated in the same conditions up to early stationary phase of growth (from 12 to 48 h depending on the bacterium). Cultures were then centrifuged (10,000 ×g, 30 min), and the supernatants were filtered (0.45 *μ*m) and kept for further analysis. MRSC was used as control supernatant.

### 2.2. Fractionation and Separation of Extracellular Proteins

Extracellular proteins were obtained from bacterial supernatants following the protocol described by Sánchez et al. [17]. Briefly, trichloroacetic acid (TCA) was added to the spent supernatants at a final concentration of 6% (w/v) and incubated ON at 4°C. Samples were then centrifuged and washed twice with ice-cold acetone (10,000 ×g, 4°C, 10 min). Pellets were dried for one hour at 37°C in a heat block, and the precipitate corresponding to 50 mL of culture was resuspended in 200 *μ*L of Laemmli buffer 5X [18] with the help of an ultrasound bath (15 min) (Ultrasons-H, JP Selecta, Spain). Samples were finally centrifuged (16,000 ×g, 21°C, 5 min) to precipitate nonsolubilized proteins and volumes of 15 *μ*L were loaded in polyacrylamide gels (12.5%) under denaturing conditions (SDS-PAGE). Prestained Page Ruler (Thermo Fisher Scientific, Madrid, Spain) was used as molecular mass marker. Extracellular proteins were separated according to Laemmli [18], in an electrophoresis buffer containing SDS (1 g/L), TRIS (3 g/L), and glycine (15 g/L) (Sigma-Aldrich) at pH = 8.7, under a constant current of 40 mA. To visualize proteins, gels were stained with colloidal Coomassie blue (GelCode Blue Safe Protein Stain, Thermo Fisher Scientific), according to the manufacturer's instructions.

### 2.3. Analysis of the Serum Reactivity to Bacterial Extracellular Proteins by Western-Blot

For detection of immunoreactive bands within the different extracellular protein extracts, 40 *μ*g of protein was separated in SDS-PAGE as described above. Proteins were transferred and immobilized onto polyvinylidene fluoride (PVDF) membranes (GE Healthcare, Madrid, Spain) for 30 min under a constant voltage of 50 V. PVDF membranes were blocked with PBS (phosphate buffered saline, Oxoid Ltd., UK) supplemented with 0.1% (v/v) Tween-20 (PBST) (Sigma-Aldrich) and with 5% (w/v) skimmed milk (PBST-L) (Oxoid Ltd.) for 3 h at room temperature. Membranes were washed twice and individual sera were diluted to 1 : 100 in PBST-L and incubated over the membranes (ON, 4°C) with slight agitation. Membranes were then washed 4 times with PBST and incubated for 1 h with a secondary antibody conjugated to horseradish peroxidase (horseradish peroxidase-conjugated anti-human IgA or IgG (Sigma-Aldrich)), diluted to 1 : 2,000 in PBST-L. For detection of immunoreactive bands, a commercial solution containing the chromogenic reagents chloronaphthol and diaminobenzidine (CN/DAB Substrate Kit, Thermo Fisher Scientific) was used.

Detection of immunoreactive bands was performed using a collection of sera and consisted of 50 samples from healthy individuals, 37 samples from individuals with CD, and 15 samples from individuals with UC. Approval for the study was obtained from the Regional Ethics Committee for Clinical Investigation (Comisión Asesora de Bioética del Principado de Asturias; Servicio de Salud del Principado de Asturias) and all determinations were performed with fully informed written consent. This collection is deposited in the “Colección del Registro Nacional de Biobancos”, Ref. C.0001263 (https://sede.isciii.gob.es/). Demographic and clinical parameters of the UC and CD patients are shown in Tables 1 and 2.

### 2.4. Purification of the Cell-Wall Hydrolase of LGG

Around one milligram of extracellular protein fraction from LGG was separated in polyacrilamide gels, as described before, using multiple wells. CWH is the only extracellular protein of LGG separated in the gel range of 70–80 kDa, so this zone was cut out of the gels with the help of the pre-stained molecular mass marker, containing a red-labelled band of 70 kDa. Gel slides were placed into dialysis membranes with a cut-off of 8 kDa and 3 mL of electrophoresis buffer were added. Dialysis membranes were set in the electrophoresis chamber and the system was maintained for one hour at a constant voltage of 170 V. Following this procedure, CWH was electroeluted from the gel slides. CWH protein, already in solution, was concentrated in a vacuum device at room temperature (Concentrator 5301, Eppendorf AG, Germany), and finally dialyzed against PBS for 24 h at 4°C (Tube-O-dialyzer devices, G-biosciences, cut-off 1 kDa). Protein concentration was determined by the bicinchoninic acid test (BCA kit, Thermo Fisher Scientific), using a series of bovine serum albumin standards.

### 2.5. ELISAs against the Cell-Wall Hydrolase of LGG

Specific IgG and IgA antibodies developed against the CWH of LGG were titrated in all the sera. Two *μ*g of CWH, purified as described above, was dissolved in PBS and coated on the surface of a 96 well plate (Nunc) 1 h at 37°C and then ON at 4°C. Wells were washed twice with 300 *μ*L of PBST and blocked with 200 *μ*L of 1% (w/v) bovine serum albumin in PBS (1 h at RT and with gentle shaking). Wells were washed twice again with 300 *μ*L of PBST and incubated with 200 *μ*L of the sera dilutions, made with PBST, for 2 h at RT. The dilutions tested were 1 : 100, 1 : 250, 1 : 500, 1 : 1,000, 1 : 2,500, 1 : 5,000, and 1 : 10,000. Wells were washed twice with 300 *μ*L of PBST and incubated with 100 *μ*L of a secondary antibody dilution in PBST (1 : 10,000) (anti-human IgG or anti-human IgA conjugated with horseradish peroxidase, Sigma-Aldrich) in PBST, for 1 h at RT. Finally, wells were washed twice with PBST and 100 *μ*L of the chromogenic substrate ultra-TMB-ELISA (Thermo Fisher Scientific) was added and incubated for 20 min at RT. Reactions were stopped with 2 M H_2_SO_4_ and the Abs_450_ of the wells measured. The titer of each serum was defined as the last dilution of serum that gave a positive reaction (Abs_450_ threshold = 0.300).

### 2.6. CWH Bioinformatic Analysis and Synthesis of a Specific Peptide

Homology searches using the CWH amino acid sequence (gi|258507319; ref|YP_003170070.1) were performed using the BlastP algorithm implemented at the National Center for Biotechnology Information (http://blast.ncbi.nlm.nih.gov/Blast.cgi) against the curated, nonredundant “nr” database. Homologous proteins present in other species belonging to the Firmicutes phylum were retrieved and aligned, and the comparative analysis allowed us to identify a specific peptide present in the CWH protein from* L. casei* subsp.* rhamnosus* that was not present in other intestinal or food bacteria species (query performed on February 24, 2014). Two mg of the specific CWH peptide (CWHp) was chemically synthesized by the company Genecust (Luxembourg), with a minimum purity of 95%.

### 2.7. CWHp against the Peptide ELISAs

Antibodies developed against the CWHp and present in the following sera C72, C65, C49, C59, P8, P39, P34, and P20 were determined by ELISA as described before. These sera were chosen as being representative for the specific CWH anti-IgA/anti-IgG values. Five *μ*g of the peptide was coated on the surface of 96-well plates (Nunc), and the following serum dilutions were tested for titration: 1 : 100, 1 : 250, 1 : 500, 1 : 1,000, 1 : 2,500, 1 : 5,000, and 1 : 10,000. The rest of the procedure was exactly as described for the CWH.

### 2.8. Statistical Methods

In order to evaluate and analyze distribution of data, the following tests were applied: the Runs test, Levène's test, and the Kolmogorov-Smirnov test. The nonparametric Kruskal-Wallis test was used for median comparison between groups (healthy controls, CD, UC, and IBD). All tests were performed using SPSS v18.0 software.

## 3. Results and Discussion

Several immunological studies have shown that the human immune system, in the framework of IBD, reacts differentially against many of the microorganisms composing our gut microbiota by producing specific IgA and IgG responses such as ASCA [19]. Experimental evidence points to extracellular proteins secreted by probiotic bacteria as molecular effectors of their beneficial effects on the human host [20–23]. Specific antigens developed against this subset of proteins, which are normally consumed with fermented and functional foods, may be related to IBD.

Susceptibility to IBD has been associated with the so-called leaky gut syndrome (LGS), in which a decrease in the gut epithelial barrier led to an increase of antigens from food and bacteria from the gut lumen leaking into the body [24]. Also, it is known that excessive bacterial translocation in CD is a key factor in the development of the disease, in which certain genetic mutations affect the autophagy pathway [25]. In fact it has been proposed that antigens belonging to commensal microbiota could interact with the immune system evoking antibody-driven inflammatory responses [26]. In a healthy gut, the immune system reacts against commensal microbiota by producing antibodies, notably secretory IgA [27, 28].

**Figure 1:**
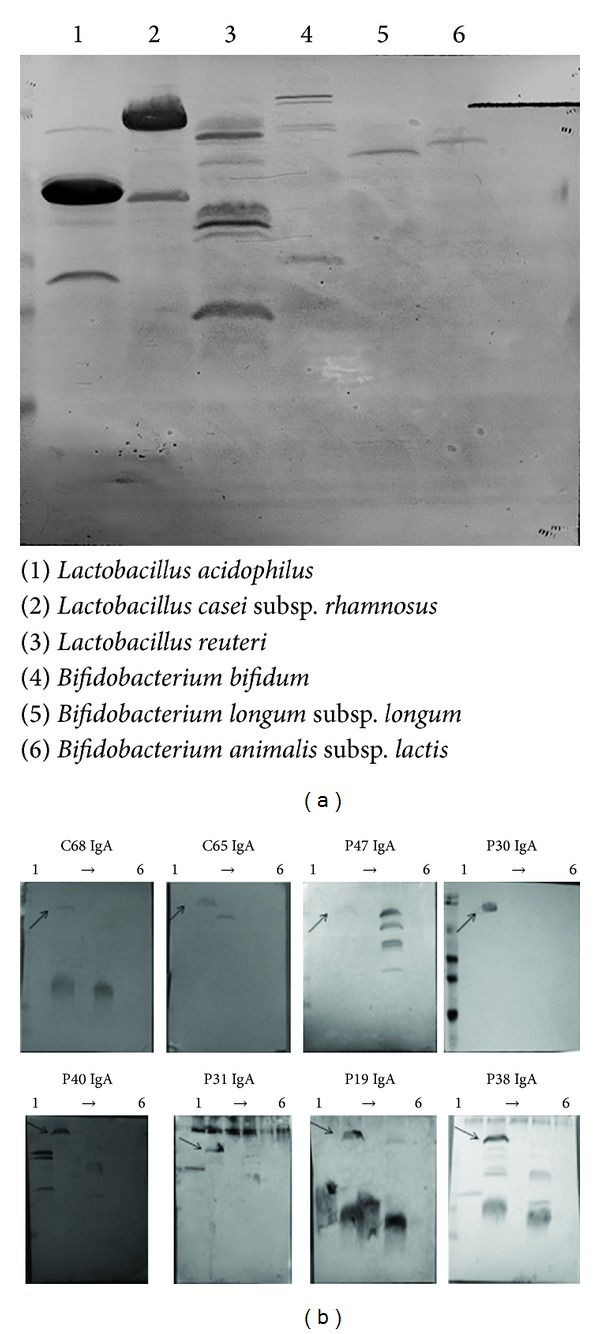
(a) Immunoreactive bands corresponding to antibodies present in the sera of healthy controls or IBD patients raised against the extracellular proteins of the bacterial strains used in this study. (b) Western blotting analysis showing different patterns of sera immunoreactivity against extracellular proteins produced by the strains used in this study (C# healthy controls; P# IBD patients). In all cases, a secondary anti-human IgA antibody, HRP conjugated, was used. The order of the lanes (lines 1 to 6) correspond to that of the SDS-PAGE.

Extracellular proteins from the bacteria strains used in this study, all of them representative of microorganisms used as probiotics or in human nutrition, were immobilized on PVDF membranes and submitted to immunoblot analysis. Incubation with pools of sera of the three different groups (HC, CD, and UC), showed the presence of different immunoreactive bands when membranes where developed using an anti-human IgA antibody conjugated with horseradish peroxidase (Figure 1(a)). The presence of strong immunoreactive bands prompted us to hybridize immobilized extracellular protein extracts using all the sera individually (Figure 1(b)). This analysis revealed that a protein in the molecular mass range of 70 kDa, produced by LGG, displayed a wide range of immunoreactivity among all the tested sera, including healthy controls and IBD patients. This protein was identified by peptide mass fingerprinting as the homologous to* L. casei* cell-wall hydrolase, also known as P-75. This is a cell-wall hydrolase able to hydrolyze muropeptides from* L. casei *[29]. In addition, CWH has an affinity for molecules present on intestinal surfaces, such as mucin and fibronectin [30]. CWH and P-40, being two of the most abundantly secreted proteins by LGG with the glyceraldehyde-3-phosphate dehydrogenase [31], accomplish different functions in gut homeostasis, such as reduction of inflammatory damages and increases of transepithelial-resistance by increasing tight-junction protein production [32, 33].

At this point, our results strongly suggested that the levels of specific anti-CWH IgAs were increased in the IBD sera. As Western-blot is very limited for antibody quantification, we purified enough amounts of CWH following the procedure described in the Material and Methods section. Once CWH was purified, and after verifying its purity by SDS-PAGE, the protein was used for the coating of 96-well plates and for quantifying the levels of specific antibodies present in the sera collection. After incubation of the different sera with the coated CWH, we used two types of secondary antibodies for detection of different antibody isotypes specifically developed against CWH, anti-human IgG, and anti-human IgA.

**Figure 2:**
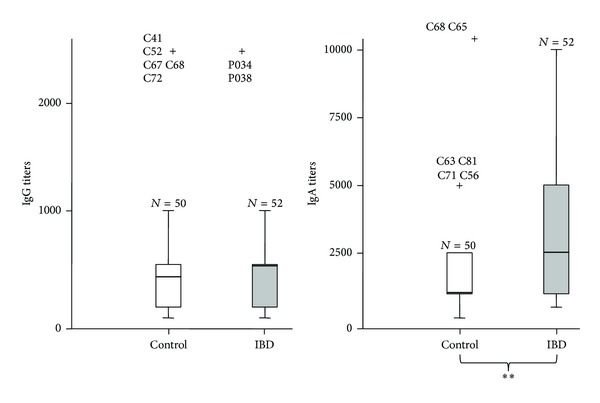
Distribution of the specific anti-CWH titers (IgG and IgA) of all the sera divided into two groups (HC: healthy controls, IBD: inflammatory bowel disease) (**P < 0.01).

**Figure 3:**
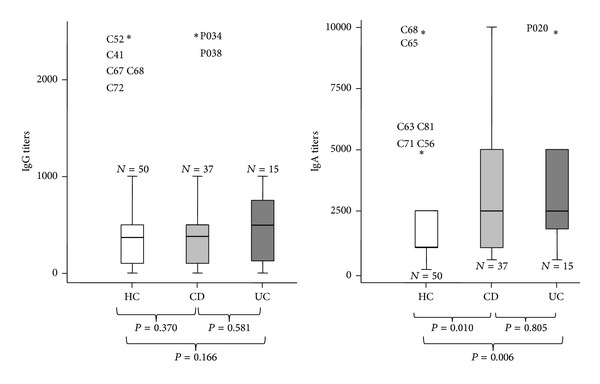
Distribution of the specific anti-CWH titers (IgG and IgA) of all the sera assayed taking into account three groups: healthy controls (HC), Crohn's disease (CD), and ulcerative colitis (UC).

While levels of specific IgGs remained constant between the two populations (healthy controls versus IBD patients) with the exception of some outlier, levels of anti-CWH IgA were significantly higher in the IBD sera (Figure 2). However, when the IBD patients were divided in CD and UC and the levels of the anti-CWH IgA were analyzed again, titers were unable to differentiate between the two conditions (Figure 3). It is known that IBD patients showing high serum reactivity against gut microbes have the most complicated disease phenotypes^13^. In this context we can mention ASCA, p-ANCA, and anti-flagellin antibodies, with the latter being even more frequent in other inflammatory disorders such as irritable bowel syndrome (IBS) [16]. In a study conducted by Furrie et al. [26], UC patients showed a higher IgG response than CD patients. Contrary to this work, no differences in the anti-CWH IgG titers were found between the IBD group and the HC group, even if IBD patients were divided into UC and CD subgroups.

In a step forward, we compared the amino acidic sequence of the CWH protein from LGG against the nonredundant protein database at the NCBI servers, in order to determine whether the anti-CWH antibodies were or were not specific to this protein. A BLAST analysis allowed us to identify a 13-mer peptide which was only present in the CWH from* L. casei* subsp.* rhamnosus* 365-VNTSNQTAAVSAS-377. Chemical synthesis of the peptide allowed us to measure the specific antibodies (anti-IgG and anti IgA) present in the different sera. In this case, we used the sera corresponding to these healthy controls/IBD patients C49, C59, C65, C72, P8, P20, P34, and P39. These sera were chosen as representative of the specific CWH anti-IgA and anti-IgG titer distribution. IgA titers against the specific CWH peptide, although lower than those measured for the whole CWH protein, were also higher in the IBD sera and lower in the healthy controls (Table 3).

Surprisingly, titers corresponding to the specific CWH-peptide IgG antibodies were zero in all the sera, suggesting that they have been developed against homologous surface proteins of other Gram positive bacteria. In this sense, presence of certain enteropathogens, notably infection by* Mycobacterium avium* subsp.* paratuberculosis*, has been proposed as a trigger factor for IBD, although controversial data have been published so far [34]. Also, increased levels of antibodies developed against proteins secreted by* M. avium *subsp.* paratuberculosis* in Japanese patients suffering from CD have been reported [35].

Why levels of IgA antibodies against extracellular proteins produced by gut bacteria are more elevated in IBD patients? It is known that increased gut permeability may correlate with IBD susceptibility/risk. This elevated passage of bacterial antigens in the intestinal mucosa may lead to a concomitant increase in the immune response through specific antibody production for compensating this failure in the barrier function of the gut epithelium [24]. In the case of familial Mediterranean fever (FMF), which is another autoimmune disease with inflammatory conditions and skin manifestations, levels of IgG antibodies against common gastrointestinal bacteria such as* Bacteroides* sp.,* Parabacteroides* sp.,* E. coli,* and* Enterococcus* sp. were increased [36].

## 4. Concluding Remarks

Our experiments showed that IgA antibodies specifically developed against an extracellular protein of* L. casei* are associated with IBD. Quantification of antibodies against extracellular proteins from food and probiotic bacteria may be helpful in the early diagnosis of IBD. Therefore, our results suggest that anti-CWH IgA levels have great potential for being used for early detection of CD and UC; however, validation of these findings in different populations of greater sample size would be required, together with the identification of other extracellular protein targets present in food and probiotic bacteria.

## Figures and Tables

**Table 1:** Demographic and clinical parameters of UC patients.

Clinical parameters	*n* = 16
Men/women	11/7
Age, mean ± sd	45.25 ± 16.89
Disease duration, mean ± sd	8.37 ± 8.57
Age at onset, mean ± sd	36.88 ± 16.79
Familial history	5 (31.3)
Location	
Extensive	9 (56.3)
Left-sided	2 (12.5)
Proctitis	5 (31.3)
Extraintestinal manifestations	
Arthritis	4 (25.0)
Dermatological affection	4 (25.0)

Values are *n* or *n* (%), unless stated otherwise.

**Table 2:** Demographic and clinical parameters of CD patients.

Clinical parameters	*n* = 37
Men/women, *n *	23/14
Age, mean ± sd	42.89 ± 12.89
Disease duration, mean ± sd	10.08 ± 8.56
Age at onset, mean ± sd	32.78 ± 12.41
Familial history	8 (21.6)
Location	
Ileum	11 (29.7)
Colon	5 (13.5)
Ileum + colon	20 (54.1)
Upper gastrointestinal	1 (2.7)
Extraintestinal manifestations	
Arthritis	14 (37.8)
Dermatological affection	9 (24.3)
Perianal disease	12 (32.4)
Fistula	15 (40.5)

Values are *n* or *n* (%), unless stated otherwise.

**Table 3:** Comparison between the titers values obtained in different sera (C#: healthy controls, P#: IBD patients) using different target proteins (CWH: purified cell wall hydrolase, CWHp: specific CWH peptide) and different secondary antibodies (Anti-human IgA and IgG). Results are expressed as the median of 3 independent measures.

Patient	Anti CWHp-IgG (antiCWH IgG)	Anti CWHp-IgA (antiCWH IgA)
C72	0 (2,500)	250 (2,500)
C65	0 (500)	500 (1,000)
P34	0 (2,500)	250 (2,500)
P20	0 (500)	1,000 (10,000)
C49	0 (250)	500 (1,000)
C59	0 (250)	500 (1,000)
P39	0 (500)	500 (2,500)
P8	0 (500)	1,000 (2,500)
